# Accelerating annotation of articles via automated approaches: evaluation of the neXtA_5_ curation-support tool by neXtProt

**DOI:** 10.1093/database/bay129

**Published:** 2018-12-14

**Authors:** Aurore Britan, Isabelle Cusin, Valérie Hinard, Luc Mottin, Emilie Pasche, Julien Gobeill, Valentine Rech de Laval, Anne Gleizes, Daniel Teixeira, Pierre-André Michel, Patrick Ruch, Pascale Gaudet

**Affiliations:** 1 Computer and Laboratory Investigation of Proteins of Human Origin Group, SIB Swiss Institute of Bioinformatics, Geneva 4, Switzerland; 2 Haute école spécialisée de Suisse occidentale, Haute Ecole de Gestion de Genève, Carouge, Switzerland; 3 SIB Text Mining, SIB Swiss Institute of Bioinformatics, Geneva 4, Switzerland

## Abstract

The development of efficient text-mining tools promises to boost the curation workflow by significantly reducing the time needed to process the literature into biological databases. We have developed a curation support tool, neXtA_5_, that provides a search engine coupled with an annotation system directly integrated into a biocuration workflow. neXtA_5_ assists curation with modules optimized for the thevarious curation tasks: document triage, entity recognition and information extraction.

Here, we describe the evaluation of neXtA_5_ by expert curators. We first assessed the annotations of two independent curators to provide a baseline for comparison. To evaluate the performance of neXtA_5_, we submitted requests and compared the neXtA_5_ results with the manual curation. The analysis focuses on the usability of neXtA_5_ to support the curation of two types of data: biological processes (BPs) and diseases (Ds). We evaluated the relevance of the papers proposed as well as the recall and precision of the suggested annotations.

The evaluation of document triage by neXtA_5_ precision showed that both curators agree with neXtA_5_ for 67 (BP) and 63% (D) of abstracts, while curators agree on accepting or rejecting an abstract ~80% of the time. Hence, the precision of the triage system is satisfactory.

For concept extraction, curators approved 35 (BP) and 25% (D) of the neXtA_5_ annotations. Conversely, neXtA_5_ successfully annotated up to 36 (BP) and 68% (D) of the terms identified by curators. The user feedback obtained in these tests highlighted the need for improvement in the ranking function of neXtA_5_ annotations. Therefore, we transformed the information extraction component into an annotation ranking system. This improvement results in a top precision (precision at first rank) of 59 (D) and 63% (BP). These results suggest that when considering only the first extracted entity, the current system achieves a precision comparable with expert biocurators.

## Introduction

Biomedical databases support many aspects of biological research, from getting basic information about a gene or a protein, to complex applications for data analysis. The usefulness of these databases critically depends on the amount of information, its correct interpretation and the regular updating of the content. For the vast majority of databases, these curatorial tasks are done manually by curators with expertise in the specific domain of interest of the database. To give an appreciation of the scope of the task, the volume of biomedical literature in PubMed, a free literature search service developed and maintained by the National Center for Biotechnology Information, currently containing 28 million citations, has increased at a sustained growth rate of ∼4% over the past 20 years (1).

It has been stated repeatedly that manual curation is inadequate to keep up with the volume of information published (for example in (2)). Meanwhile, no fully automated tools have been successfully implemented in the annotation workflow of major databases. The essential features required for a complete or at least partial replacement of manual curation include accurate prioritization of the literature to serve database-specific curation tasks, correct detection of bioentities (named-entity recognition) as well as recall and precision rates approaching manual curation. Moreover, since best practices in curated databases require the assignment of unique identifiers to entities derived from biomedical ontologies, automated tools should be able to convert natural language into these controlled languages. Tools able to perform those tasks can be used to perform literature triage, bioentity identification and normalization, relationship extraction [typically between a gene product and a disease (D) or a biological process (BP), for instance] and association of supporting evidence qualifiers (3). These tools would facilitate and accelerate the curation process, hence improving its cost-effectiveness and throughput.

The ideal tool for retrieving biomedical information would display a user-friendly interface, provide a powerful search tool from databases containing up-to-date biomedical data, allow a search within specific sections of articles, highlight terms of interest, display results that could be filtered and ranked, create annotations and respond fast following the request. Existing text-mining tools exhibit some of these features but none have all the required functionality, as we show in our analysis of currently available text-mining-supported curation tools (Table 1). We assessed Textpresso Central (4), PubMed (5), NextBio, PolySearch (6), GoPubMed (7) and PubTator (8) and evaluated all parameter listed in Table 1. We also looked at the workflow of other text-mining tools, such as Argo (9–12), Egas (13), EXTRACT (14), MetastasisWay (15), Ontogene (16) and RegulonDB (17), but because they are dedicated to specific biomedical fields (and not appropriate for our use cases), we didn’t include them in our comparative study. The functionalities important to the curation workflow must be close in quality to that of manual annotation. However, direct comparison is not always possible as automatic systems exhibit characteristics that do not align one-to-one with curation tasks as performed by humans. More importantly, the digitalization of curation workflows may require to challenge existing end-users’ practices and well-established workflows (18); data stewardship and capture need revision in order to also keep track of materials rejected by biocurators (wrong annotations, irrelevant articles etc.). Nevertheless, for the annotations proposed by the system, a precision of 60–70% seems a minimal—yet demanding—target to meet the curators’ expectations. Similar quantitative targets also apply to triage tasks. Considering that a 100% manual triage is not achievable, any improvement over existing tools is welcome. Indeed, triage tasks are a bottleneck and cannot be performed without using general-purpose search engines such as PubMed or Europe PubMedCentral (PMC).

**Table 1:** Comparison of some existing text-mining tools

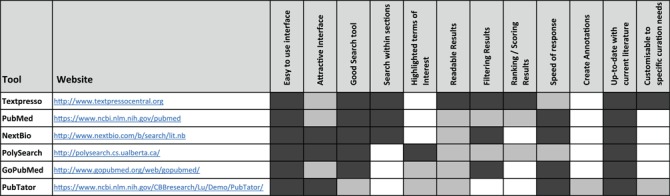


The performance of the main parameters important for the curation workflow is indicated by the degree of shading: white means feature not available; light grey, medium performance; and dark gray, very good performance.

neXtProt (19) is a knowledgebase focused on human proteins, which complements UniProtKB (20) by extending the content and tools, supporting use cases specifically relevant to human proteins. neXtProt manually annotates various aspects of protein function, variants and phenotypes (19, 21). To do this, we have developed a curation tool, the BioEditor, that allows curators to capture biomedical data. Annotations are structured in triplets, in accordance with the neXtProt BioEditor annotation data model. The triplets are composed of a subject (the protein being annotated); an object describing a gene ontology (GO) term, a D, an interaction partner etc.; and a relation describing how the subject and the object are related.

We have developed an automatic article-processing tool that addresses our specific curation needs, neXtA_5_ (22, 23). neXtA_5_ provides a search engine coupled with an annotation system, directly integrated into the workflow of curators. Thus, neXtA_5_ assists curation with specific modules optimized for the various curation tasks: document triage, entity annotation and relationship extraction. The tool performs literature retrieval and prioritization and creates annotations. The curator queries the system by entering a human gene name and an axis of interest. For the purposes of this study, two axes were evaluated: GO BP as well as Ds. The system returns a ranked list of abstracts and concepts for the relevant axis for each of the papers. The curator can select the relevant articles/gene/concept combination and validate/refine/reject annotations proposed by the system.

In previous work, we have optimized the ranking algorithm of neXtA_5_ for the triage task. The tool exhibits significant improvements of 191–261% compared to PubMed (22, 23). The present article describes the testing and evaluation of neXtA_5_ by expert curators. To evaluate the accuracy and performance of neXtA_5_, we submitted specific requests and then compared the results obtained from manual curation to the results given by the neXtA_5_ application. The analysis is focused on the usability of neXtA_5_ on two types of annotations: BPs and Ds, respectively defined as GO concepts (24, 25) and National Cancer Institute thesaurus (https://ncit.nci.nih.gov/). We have evaluated the relevance of the papers proposed as well as the recall and precision of the concepts extracted.

## Methods and results

### neXtA_5_ software infrastructure

The neXtA_5_ system was developed with Java/JavaScript technologies to improve the scientific literature curation process as it is currently performed with neXtProt.

#### Publication retrieval and concept extraction

SIB Text Mining houses the complete MEDLINE collection locally, updated on a weekly basis, in an information system named BioMed, that pre-indexes the collection using the Terrier and ElasticSearch platforms (26, 23) according to vocabularies relevant to the axes of interest. Again, here we focused on GO BP and Ds. BioMed services support the maintaining of several premier molecular biology databases, including Europe PMC’s SciLite or UniProt’s UPCLASS (27–29). Indexed papers are analyzed and concepts from the ontology of interest are extracted and stored in the BioMed database, as well as human gene names obtained from the neXtProt application programming interface (API). Once the information is stored, BioMed applies a combination of weighting schemas, which includes a vector space model representation (30), and the Okapi BM25 scoring function, which was tuned and tested during Text Retrieval Conference (TREC) competitions (31). This results in two outputs: (i) a ranked list of abstracts and (ii) for each abstract, a ranked list of concepts for the axis of interest. The ranking function is described in a previous publication (22).

#### Document prioritization

The list of documents provided by the search engine is further ranked with a score based on a linear combination of factors; each of the search axis was tuned specifically to fit the curation model of neXtProt curators as detailed in (22, 23). This final score is calculated on the basis of the search engine score, combined with the range of concepts found in the paper and the term frequency–inverse document frequency (TF–IDF).

**Figure 1:**
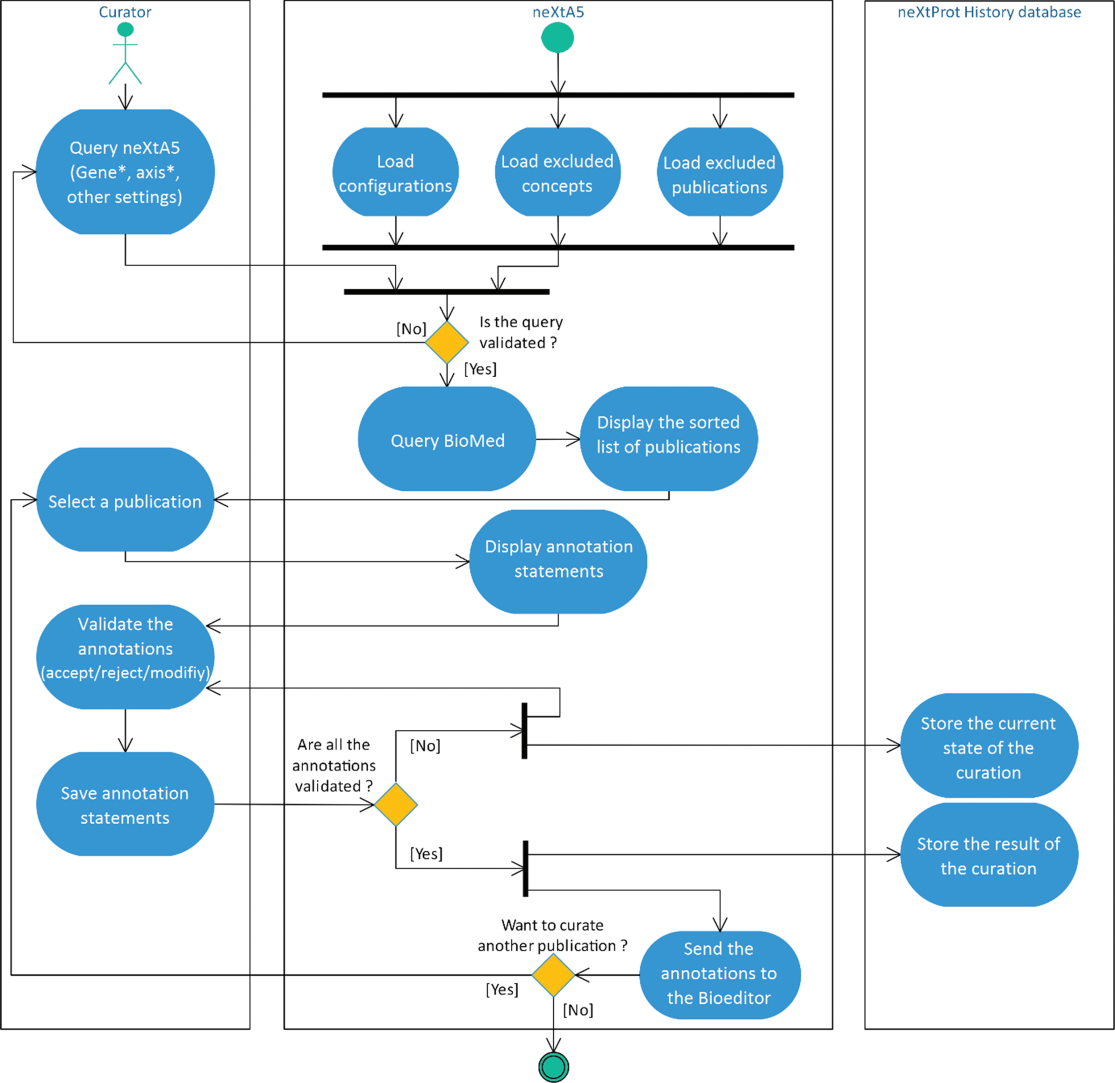
Activity diagram of the literature curation process using neXtA_5_.

#### User interface

We have implemented a web-based curation interface that connects the BioEditor curation database with a set of APIs. The first screen is dedicated to the user input, with customized intake fields to refine the original query. The second panel displays the result of the triage function, with the final score granted to each document. Finally, in a third screen, a list of automatically generated annotations is proposed for each document. Each entry can be accepted as it stands, rejected or modified as needed. At the end, the curator can submit the annotation to the BioEditor. The work can also be saved at any time and completed subsequently. Indeed, the graphical user interface (GUI) is also linked to a historical database that keeps track of the curation process and results, which can also serve to set out a relevance feedback. This history enables the system to remember every processed publication and remove them from upcoming searches (using the same query).

### neXtA_5_ user interface

The workflow of the neXtA_5_ curation-support tool is shown in Figure 1.

**Figure 2:**
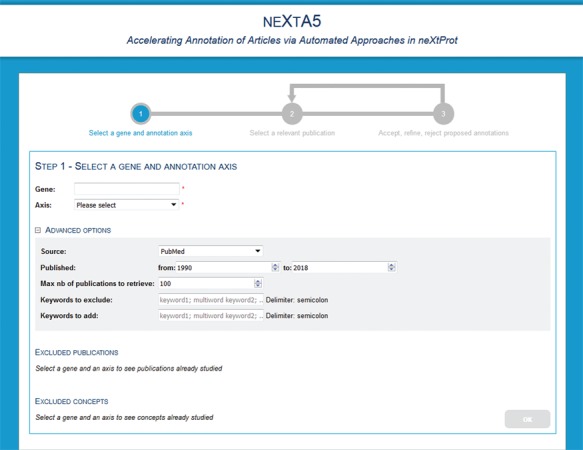
neXtA_5_ user interface for query page.

The neXtA_5_ user interface is designed to assist specific biocuration tasks (Figure 2). The user performs a query, which is a gene name and an annotation axis. Additional features include the ability for users to exclude specific references that will not be retrieved by the system (e.g. publications that were previously processed or publications of low interest). Users can also provide keywords that must be ‘excluded’, for instance because they result in too many false positives, or ‘added’, in which case they will receive more weight during the ranking step, for the ranking. Finally, advanced options allow the user to restrict the search based on a range of publication dates, the maximum number of publications to retrieve.

The output of the query is a list of publications, ranked according to the relevance score developed in (22–23). The list displays relevant information about the publication, including the PMID, the title, the year of publication, the relevance score and the annotation status. Different annotation statuses are possible: ‘not done’, ‘partial’ (when some but not all the annotations proposed by the system have been reviewed by the curator) or ‘completed’ (when every automatic annotation has been manually reviewed).

**Figure 3:**
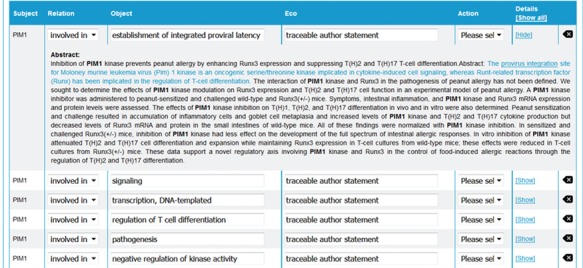
neXtA_5_ user interface for curation. From the abstract of an article, neXtA_5_ extracts relevant concepts and displays a list of potential annotations. Here, the annotations related to PIM1 for the BPs and extracted from the abstract of (32) are shown.

From this ranked list, the curator can select a paper to curate that opens another page in the user interface displaying the list of potential annotations identified by neXtA_5_. The potential annotations are presented in table form, showing the subject (which corresponds to the protein of interest), the relation, the object (concept) and the evidence code (Eco). For each annotation, when the user clicks on the ‘Show’ button (in the ‘Details’ column on the right), the abstract appears, highlighting the sentence from which the annotation was derived in blue and underlining the concept (Figure 3). Here, three operations are possible, from a pull-down menu in the ‘Action’ column; the curator can accept, modify or reject the annotations created by neXtA_5_. The curator can also change the relation linking subject and object as well as the Eco (currently these are set to default values in the interface); however, changes in the relation or the Eco does not impact the type of action; if the concept was not changed, then the annotation is considered as ‘accepted’.

### neXtA_5_ usability study

To evaluate the usability of neXtA_5_ as a curation support system, we measured the recall and precision of the annotations proposed by the system as compared to manual curation. The precision corresponds to the fraction of relevant instances among the retrieved instances, while recall is the fraction of relevant instances that have been retrieved over the total number of relevant instances. Here, ‘instances’ can correspond to either documents or concepts.

Four experienced curators from the neXtProt team reviewed the neXtA_5_ output. The evaluation focused on neXtA_5_ annotation for 12 different proteins: CDK2 (NX_P24941), CSK (NX_P41240), FYN (NX_P06241), IRAK4 (NX_Q9NWZ3), LRRK2 (NX_Q5S007), LYN (NX_P07948), PIM1 (NX_P11309), RIPK2 (NX_O43353), SGK1 (NX_O00141), STK11 (NX_Q15831), SYK (NX_P43405) and ZAP70 (NX_P43403). The proteins were selected on the basis of having sufficient literature to allow proper evaluation of the system, i.e. >100 papers in a PubMed search, while avoiding the gene normalization problem, i.e. the gene name is not used as a synonym for another gene or as an acronym for a term used elsewhere in the literature. Example of proteins we avoided includes BTK (used in orthopedic papers as an acronym for ‘below the knee’) and ABL1 (used for ABL1 and ABL2 in older literature). The latter could have been controlled using the date range, while the former can be handled by excluding the word ‘knee’. Having a certain number of different targets ensure that we cover a wide range of biological research areas, to increase the number of distinct concepts reported in the literature. This was aimed to control for biases, for example in the concept extraction step (as certain concepts have labels that are more difficult to extract by automated tools) and in the gene name extraction step (certain genes may have an abnormally high rate of false positives or false negatives, for example if a synonym is shared with another gene name or a concept or if the main gene name is not widely used in the literature).

Moreover, we ensured that each abstract was reviewed by two different curators, so as to have a measure of confidence of the evaluation of the annotations proposed by the automatic system, the rational being that when two curators do not agree, an error by neXtA_5_ should be less penalized.

**Table 2:** Semantic classification of concepts annotated by the curators or proposed by neXtA_5_

**Semantic classification**	**GO terms**
1	Reactive oxygen species biosynthetic process
	Reactive oxygen species metabolic process
	ROS generation
2	S phase
	DNA replication
	Regulation of cell cycle
3	Autophagy
	Autophagosome assembly
	Autophagosome formation

### Setting the baseline: inter-curator agreement

Since curation is a subjective process to some extent, before comparing neXtA_5_’s performance as evaluated by curators, we determined the agreement between different curators for the tasks we evaluated for neXtA_5_.

#### Strategy for assessing agreement with respect to concept extraction

Since the BP branch of the GO has nearly 30 000 classes, the selection of 2 different terms by 2 curators does not automatically imply a disagreement. The evaluation must take into account how related two terms are to decide whether two curators (or a curator and the automatic system) recognized a similar concept or not. To do this, we manually reviewed all annotated concepts (both by curators and by neXtA_5_) for all abstracts and manually assigned each concept to a semantic class, numbered from 1 to *n* for each abstract. This is illustrated in Table 2. In this example, curators identified 9 different GO terms, which we classified into three semantic classes, labeled 1, 2 and 3. Concepts falling in the same semantic class were considered equivalent in our evaluation.

**Figure 4:**
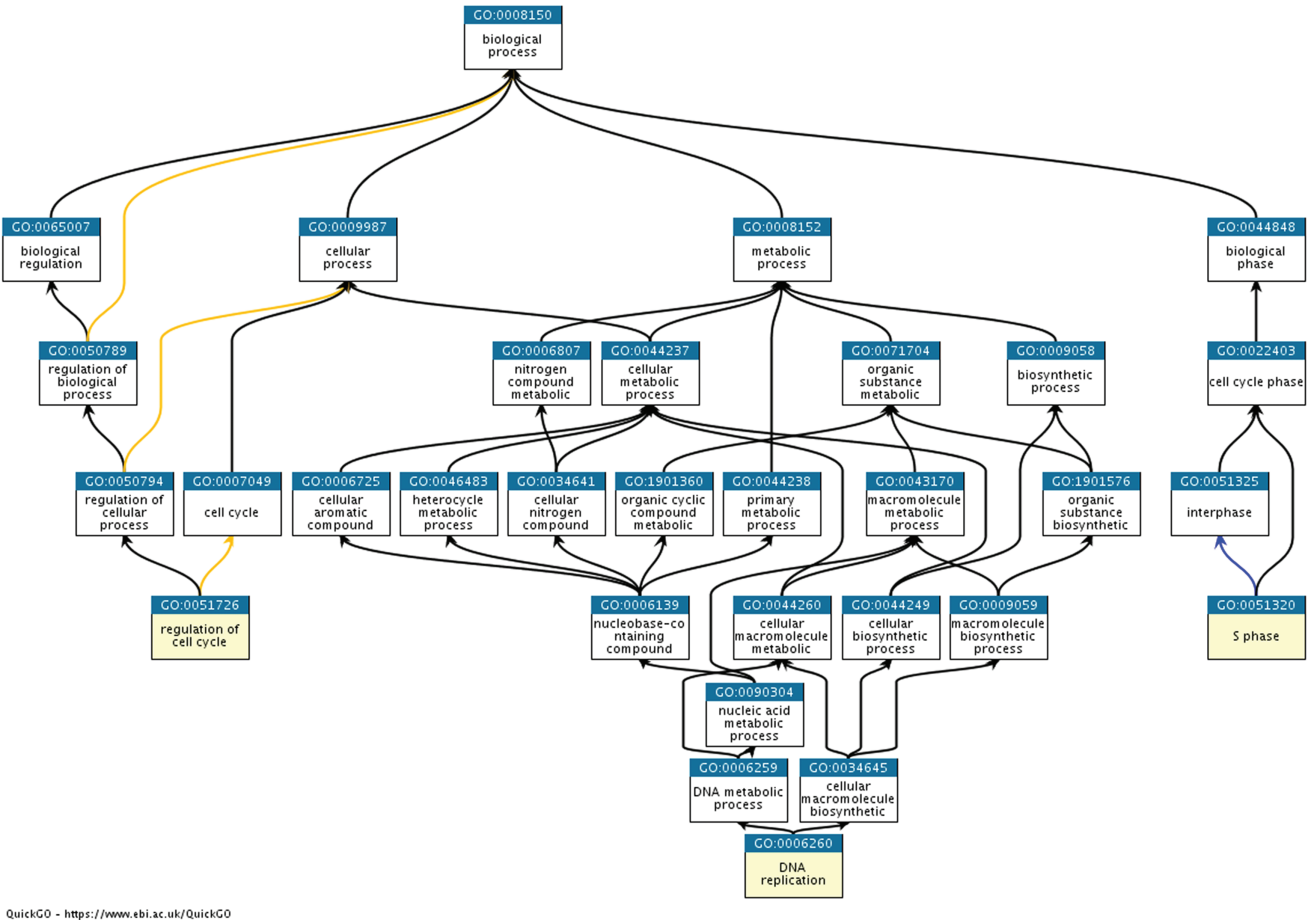
Ancestor charts of the GO terms from semantic classification 2, shown in Table 2 [S phase (GO:0051320), DNA replication (GO:0006260) and regulation of cell cycle (GO:0051726)], using https://www.ebi.ac.uk/QuickGO/.

Here, we have decided to use a manually semantic classification approach rather than using the hierarchical structure in the GO. While the hierarchy of the GO could be used for this purposes (as in cases 1 and 3 in Table 2), in other cases GO terms that represent the same experiment correspond to completely different areas of the tree, as shown in case 2 Table 2. We have grouped the three GO terms S phase (GO:0051320), DNA replication (GO:0006260) and regulation of cell cycle (GO:0051726) into the same semantic classification group by manual classification, whereas these concepts belong to three different branches of the GO, as shown in Figure 4.

#### (i) Inter-curator agreement test for precision of document retrieval

**Figure 5:**
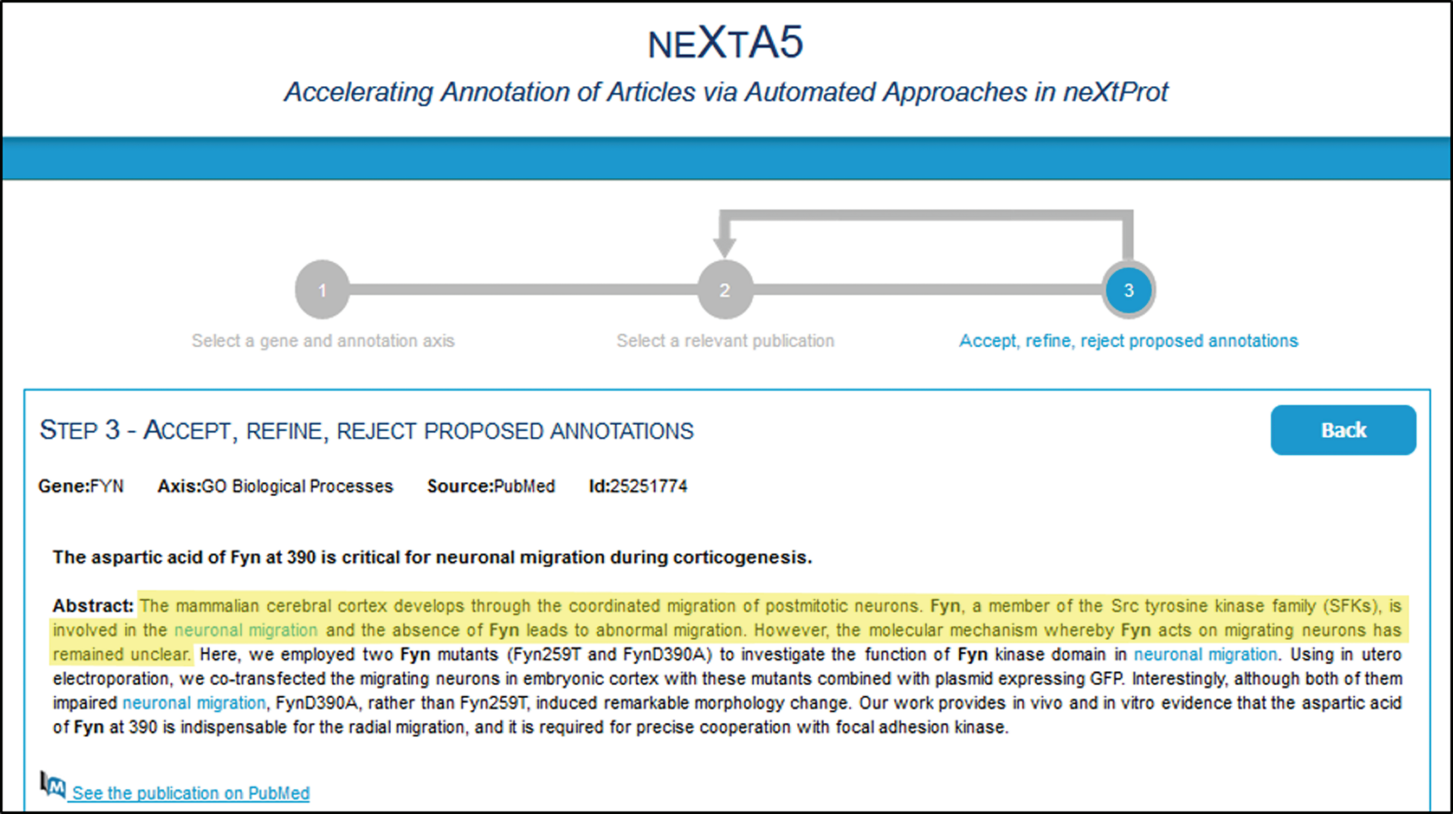
neXtA_5_ user interface for curation. One of the guidelines for the curators to select relevant documents was to not consider statements from titles and from the introductory part of the abstract. Here, the introduction of the abstract of (37) related to FYN function (BP axis) is highlighted in yellow.

We first evaluated the inter-curator agreement with respect to the relevance of abstracts proposed by neXtA_5_, the so-called literature triage task. For this task, we determined the fraction of the first top-ranking 20 papers proposed by neXtA_5_ that were deemed relevant by both curators (assessed by whether or not they had identified relevant concepts in the abstract). The criteria for selecting an abstract as relevant for annotation were that it had information indicating that there was data in the full text paper relevant to the axis of interest. To exclude papers with general statements (rather than actual data), we specified the following guidelines: exclude statements from titles and from the introductory part of the abstract (highlighted in Figure 5); and do not capture any ‘hypothesis’ type information, such as ‘We hypothesized that the protein X performs process Y.’ Examples of such sentences include ‘Since activation of Ras oncogenes is a common oncogenic event leading to the activation of multiple effector pathways, we explored if Ras could induce Fyn expression.’ (33); ‘The fact that IRAK4, another IRAK family member necessary for the IL-1 pathway, is able to phosphorylate IRAK in vitro suggests that IRAK4 might be the IRAK kinase.’ (34); ‘The mechanism of activation for IRAK4 is currently unknown, and little is known about the role of IRAK4 kinase in cytokine production, particularly in different human cell types.’ (35); ‘In this study, we analyzed the relative PTPN22 and CSK expression in peripheral blood from 89 RA patients and 43 controls to determine if the most relevant PTPN22 (rs2488457, rs2476601 and rs33996649) and CSK (rs34933034 and rs1378942) polymorphisms may influence on PTPN22 and CSK expression in rheumatoid arthritis (RA).’ (36).

Of the 12 proteins, a total of 242 abstracts were analyzed for each axis (for 12 targets, we expected to analyze 240 abstracts; however, in some cases abstracts with the same score were presented in a different order, which led to the annotation of 2 additional abstracts). As shown in Table 3, in 83% of cases for BP and in 80% of cases for D, both curators made the same decision with respect to the relevance of an abstract for the axis of interest.

**Table 3:** Inter-curator agreement analysis

	**BPs**		**Ds**	
Papers accepted by both curators	162	**67%**	152	**63%**
Papers rejected by both curators	39	**16%**	48	**17%**
Papers rejected by just one curator	41	**17%**	42	**20%**
Total papers analyzed	**242**		**242**	

#### (ii) Inter-curator agreement test for precision of concept retrieval

The precision of concept retrieval corresponds to the number of relevant terms extracted in each document. We assessed this by determining the rate at which both curators extracted the same concepts from an abstract. Again, specific curation guidelines were given: when similar descriptors are proposed, use the most accurate one, i.e. choose preferentially the child term than the parent term (for example, reject the annotation suggesting ‘Neoplasm’ when ‘Ovarian carcinoma’ is also mentioned in another annotation); annotations describing techniques (such as ‘immunohistochemistry’) are acceptable as indication of experimental data in the full text paper; and annotations describing negative evidence are included as relevant for annotation. If a concept was modified from the original concept, it had to be within the same branch of the ontology.

**Figure 6:**
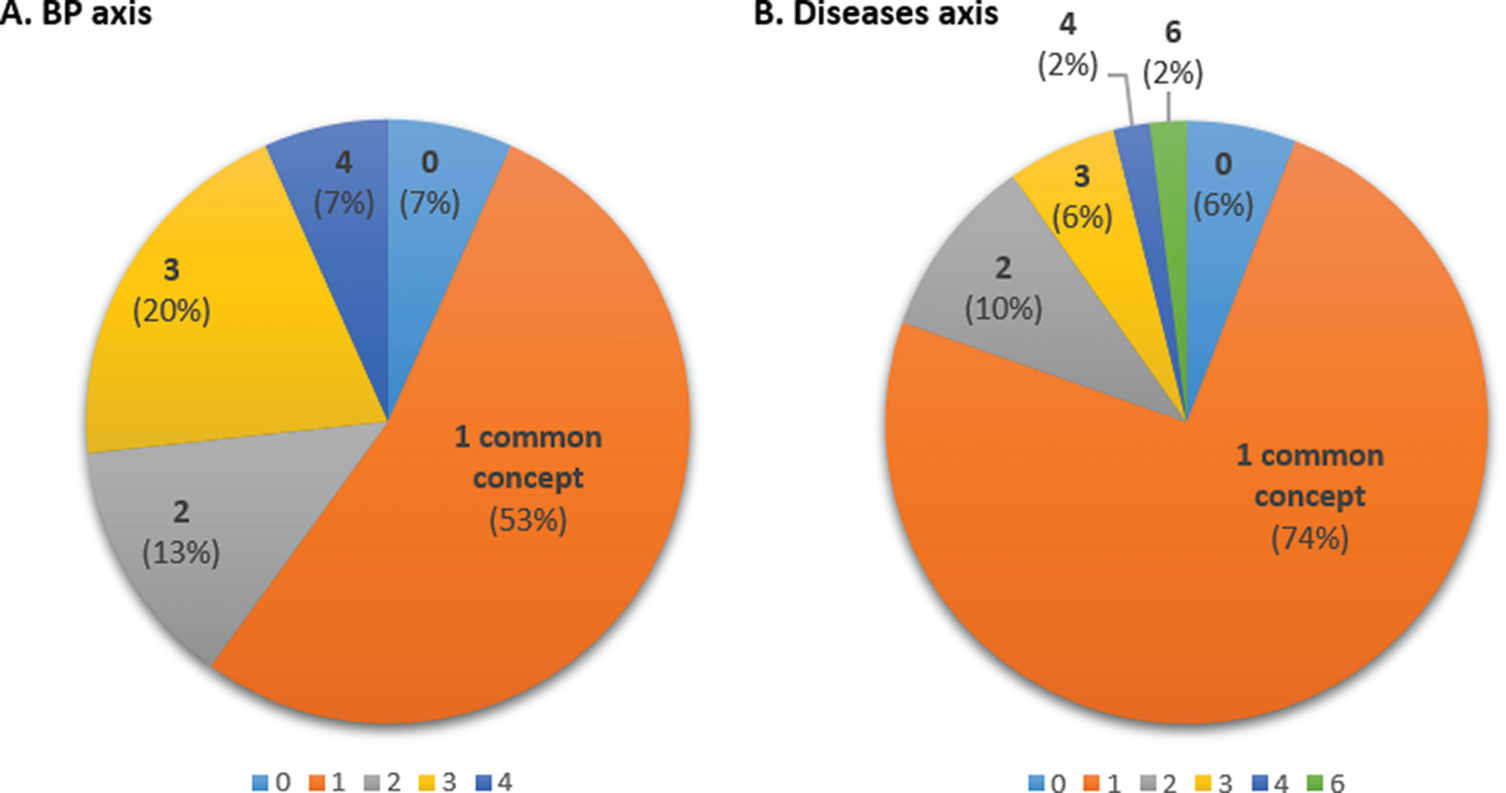
Inter-curator agreement with respect to concepts in BP (A) and D (B) axes showing the proportion of common concepts found by both curators. The number indicated is the number of common concepts identified by both curators (0–4 for BP; 0–6 for Ds).

For this task, 45 abstracts of the BP axis and 51 abstracts of the D axis were annotated by two curators with BP and D terms, respectively (while the expected number of annotated papers for this task is 48, the actual number varies because the papers chosen by different curators for annotation may differ). This corresponds to a minimum of four abstracts by curator and by protein, with a few additional abstracts to ensure that at least two curators reviewed each abstract (the additional abstracts correspond to cases where curators made different decisions with respect to the relevance of an abstract for an axis). For the 45 abstracts annotated for the BP axis by both curators, at least 1 common term was found in 42 abstracts (93% of abstracts, Figure 6A). The overall average inter-curator agreement rate with respect to concepts, i.e. the average proportion of concepts annotated by both curators relative to all concepts found by either curator, was of 60%. For the D axis, out of the 51 abstracts annotated by both curators, the 2 curators found at least 1 common term in 48 abstracts (94% of abstracts; Figure 6B). The overall average inter-curator agreement rate with respect to concepts was of 87%.

Hence, the inter-curator agreement is ~80% with respect to relevance of abstracts, regardless of the axis (Table 3), and curators find at least 1 common concept in over 90% of the abstracts (Figure 6). On average, 60% of the concepts in an abstract were identified by both curators for BP and 87% for Ds. This may reflect the greater complexity of GO compared to D terminology, which likely hampers annotation consistency.

### neXtA_5_ evaluation

We then evaluated the precision and the recall of the neXtA_5_ system. We evaluated the precision both at the level of the document retrieval and information extraction and the recall (as compared) with the manually extracted terms (as the set of expected true positives).

#### (i) neXtA_5_ precision for document retrieval

Using the data from task (i) for inter-curator agreement, we can derive the fraction of the abstracts retrieved by neXtA_5_ and that curators assessed as relevant for the axis of interest. We find that both curators agree with neXtA_5_ for 67% of the abstracts suggested in the BP axis and for 63% of the abstracts in the D axis. Moreover, for 15% of the abstracts, both curators judged that the abstract was not relevant for the axis of interest (Table 3).

#### (ii) neXtA_5_ precision for information extraction

To determine the fraction of relevant concepts that neXtA_5_ retrieved, we manually evaluated each of the annotations proposed by neXtA_5_ for the 20 first abstracts, for each of the 12 target proteins (in cases where all concepts were rejected, additional abstracts were annotated until we reached 20 evaluated abstracts). Again, each abstract was evaluated independently by 2 curators, for a total of 254 abstracts. From these 254 abstracts, a total of 3175 annotations were proposed by the neXtA_5_ system. For the BP axis, curators approved or modified the proposed descriptor (a modification is a change of term within the same branch of the GO) for 35% of the terms; hence, 65% of the descriptors were considered as non-relevant. For the D axis, curators approved or modified the proposed descriptors for 25% of the cases and rejected 75% of the descriptors (Table 4).

**Table 4:** Precision analysis for BP and D axes

	**Total number of descriptors analyzed**	**Accepted**	**Modified**	**Rejected**	**Precision**
BP	3175	699 **(22%)**	413 **(13%)**	2061 **(65%)**	**35%**
Ds	4967	1094 **(22%)**	146 **(3%)**	3727 **(75%)**	**25%**

**Table 5:** Average number of terms found by curators (common terms and total terms) and by neXtA_5_ for BP and D axes

	**BPs**	**Ds**
Number of concepts identified by at least one curator and neXtA5	1.1	1.2
Manual curator (average number of concepts/papers)	2.4	1.5
neXtA5 (average number of concepts/papers)	6.2	6.0

### neXtA_5_ recall for annotations

To assess recall, curators manually extracted descriptors (independent of the neXtA_5_ information extraction module) from the first 4 abstracts for each of the 12 target proteins, as described in task (ii). Again, two curators performed the task for each abstract. We evaluated neXtA_5_ with two different criteria: (i) based on the descriptors only identified by both curators or (ii) based on the descriptors identified by either curator. That latest assessment is the best evaluation for an automated system; if a descriptor is identified manually, regardless of whether this assignment may be disputable, we don’t expect an automatic system to be capable of such nuanced judgement.

For the BP axis, neXtA_5_ successfully identified 27% of the descriptors found by both curators and 36% of the terms identified by either curator (Supplementary Data Table 1). For the D axis, neXtA_5_ identified 42% of the terms found by both curators and 68% of the terms identified by either curator.

## Discussion

### Improvement of the manual annotation

Our results show an inter-annotator agreement (IAA) of ~80% with respect to relevance of abstracts, regardless of the axis (Table 3), and curators found at least 1 common concept in over 90% of the abstracts (Figure 6). There is little data in the literature where inter-curator agreement was evaluated, so it is difficult to judge whether this is expected. A recent study, showing the mining of clinical attributes of genomic variants using Egas, a web-based platform for text-mining-assisted literature curation, presented an overall IAA of 74% (13), while 2 other studies investigating the text-mining assisted biocuration workflows in Argo exhibited an IAA of 68.12% or varying between 67% and 84% (9, 10). Looking at some events of divergent decisions by the two curators, it seems that in most cases there was a drift from the curation guidelines and that if we return to the guidelines we can more often agree on the decision.

### Performance of neXtA_5_

We have developed neXtA_5_, a system that enhances the biocuration workflow by prioritizing research articles for specific tasks, and evaluated its performance with respect to document triage, precision and recall compared with manual annotation. These parameters are essential to develop a tool that can be used in the daily workflow of curated biological databases. We evaluated the effectiveness of the system to support the curation of GO BPs and Ds.

With respect to document retrieval, neXtA_5_ proposes ~15% of documents that are not relevant for the task at hand. This is quite acceptable, given that neXtProt curators routinely use PubMed to retrieve literature, which returns a much higher fraction of non-relevant documents, because it does not allow to specify a general domain of interest but only keywords. Moreover, this 15% is also similar to the rate at which curators disagree with each other with respect to the relevance of a document (17–20%; Table 3), thus suggesting that the current triage effectiveness is approaching a theoretical upper bound.

For the concept extraction task, neXtA_5_ had a precision rate of 35% for BP and 25% for D and a recall rate of 27% for BP and 42% for D. It must be noted that neXtA_5_ retrieves 2.6 times more descriptors compared to curators in the BP axis (Table 5). Indeed, neXtA_5_ finds an average of 6.2 concepts per abstract for the 45 abstracts annotated by both curators for the recall test, while curators find 2.4 terms and 1.1 common terms on average. In the D axis, neXtA_5_ finds an average of 6 concepts per abstract for the 45 abstracts annotated by both curators, while curators find 1.5 terms and 1.2 common terms on average. Therefore, neXtA_5_ finds four times more concepts than curators for the D axis. This high level of identified descriptors contributes to the low precision rate of neXtA_5_.

While the precision and recall performance do not yet allow for completely automated annotation, the fraction of relevant terms certainly makes the system a valuable enhancement to manual curation tasks.

## Potential improvements of neXtA_5_

While doing the evaluations, and based on their extensive experience in annotation, we noticed some recurring issues that should be addressed to enhance the performance of neXtA_5_.

### Heterogeneity of neXtA_5_ concept extraction by annotation target

We noticed significant heterogeneity in the precision of concept extraction among the different targets. For instance, in the BP axis, only 17% of the terms proposed for ZAP70 by neXtA_5_ were accepted or modified by the curators compared to 49% of the terms proposed for LRRK2 (Table 6).

**Table 6:** Precision analysis for BP (A) and D (B) axes

**A**	**BPs**			**B**	**Ds**	
	**Number of terms analyzed per protein**	**Precision**			**Number of terms analyzed per protein**	**Precision**
LRRK2	247	**49%**		LYN	398	**41%**
SGK1	301	**43%**		SYK	570	**36%**
SYK	262	**40%**		ZAP70	250	**31%**
IRAK4	236	**39%**		PIM1	444	**30%**
LYN	265	**38%**		FYN	402	**23%**
FYN	343	**36%**		RIPK2	194	**23%**
PIM1	327	**35%**		IRAK4	351	**22%**
CDK2	333	**33%**		CDK2	504	**21%**
RIPK2	145	**32%**		LRRK2	452	**19%**
CSK	318	**29%**		SGK1	491	**19%**
STK11	156	**27%**		STK11	635	**18%**
ZAP70	242	**17%**		CSK	276	**18%**

This discrepancy might be due to synonyms that cause problems (formation, growth etc.), terms that are too vague (signaling, signal transduction, signaling cascade, regulation, carcinogenesis, tumor, autoimmune D etc.), technical terms (RNA interference, RNAi, knockout mice etc.) or non-relevant terms for the axis of interest (pathogenesis, memory, methylation, phosphorylation, localization, point mutations, gene variant, accumulation, sensitivity etc.; Table 7).

**Table 7:** List of rejected terms by the curators in BP (A) and D (B) axes

Table 7A List of rejected terms by the curator in biological process axis									
**Unique concept**	**Proposed concept**	**Proposed synonym**	**Rejected**		**Modified**		**Accepted**		**Total**
GO:0023052	Signaling	Signaling	154	**56%**	63	**23%**	56	**21%**	273
GO:0032502	Developmental process	Developmental process	112	**86%**	13	**10%**	5	**4%**	130
GO:0065007	N/A	Biological regulation	110	**81%**	26	**19%**	0	**0%**	136
GO:0016310	Phosphorylation	Phosphorylation	110	**48%**	55	**24%**	66	**29%**	231
GO:0007165	Signal transduction	Signal transduction	90	**76%**	11	**9%**	17	**14%**	118
GO:0006351	Transcription and DNA-templated	Transcription and DNA-templated	88	**79%**	6	**5%**	18	**16%**	112
GO:0009058	Biosynthetic process	Biosynthetic process	83	**80%**	19	**18%**	2	**2%**	104
GO:0040007	Growth	Growth	74	**80%**	16	**17%**	2	**2%**	92
GO:0010467	Gene expression	Gene expression	55	**76%**	1	**1%**	16	**22%**	72
GO:0006915	Apoptotic process	Apoptotic process	44	**52%**	4	**5%**	36	**43%**	84
GO:0009405	N/A	Pathogenesis	42	**100%**	0	**0%**	0	**0%**	42
GO:0051726	Regulation of cell cycle	Regulation of cell cycle	40	**82%**	2	**4%**	7	**14%**	49
GO:0007049	Cell cycle	Cell cycle	37	**65%**	4	**7%**	16	**28%**	57
GO:0006954	Inflammatory response	Inflammatory response	36	**62%**	2	**3%**	20	**34%**	58
GO:0006283	Transcription-coupled nucleotide-excision repair	TCR	34	**77%**	9	**20%**	1	**2%**	44
GO:0016246	N/A	RNA interference	31	**100%**	0	**0%**	0	**0%**	31
GO:0008283	Cell proliferation	Cell proliferation	31	**60%**	1	**2%**	20	**38%**	52
GO:0009056	Catabolic process	Catabolic process	26	**59%**	10	**23%**	8	**18%**	44
GO:0033673	N/A	Negative regulation of kinase activity	26	**100%**	0	**0%**	0	**0%**	26
GO:0051179	Localization	Localization	24	**69%**	10	**29%**	1	**3%**	35
GO:0008152	Metabolic process	Metabolic process	22	**88%**	0	**0%**	3	**12%**	25
GO:0016049	Cell growth	Cell growth	21	**81%**	1	**4%**	4	**15%**	26
GO:0045087	Innate immune response	Innate immune response	19	**76%**	0	**0%**	6	**24%**	25
GO:0001816	Cytokine production	Cytokine production	17	**52%**	1	**3%**	15	**45%**	33
GO:0008219	Cell death	Cell death	16	**57%**	2	**7%**	10	**36%**	28
GO:0006412	Translation	Translation	16	**53%**	1	**3%**	13	**43%**	30
GO:0042110	T cell activation	T-cell activation	14	**70%**	0	**0%**	6	**30%**	20
GO:0051320	S phase	S phase	13	**46%**	5	**18%**	10	**36%**	28
GO:0046903	Secretion	Secretion	13	**50%**	7	**27%**	6	**23%**	26
GO:0006914	Autophagy	Autophagy	13	**65%**	0	**0%**	7	**35%**	20
GO:0030154	Cell differentiation	Cell differentiation	12	**86%**	2	**14%**	0	**0%**	14
GO:0032259	N/A	Methylation	12	**100%**	0	**0%**	0	**0%**	12
GO:0006260	DNA replication	DNA replication	11	**55%**	0	**0%**	9	**45%**	20
GO:0009293	N/A	Transduction	11	**79%**	3	**21%**	0	**0%**	14
GO:0006810	N/A	Transport	11	**100%**	0	**0%**	0	**0%**	11
GO:0046960	Sensitization	Sensitization	11	**92%**	0	**0%**	1	**8%**	12
GO:0016311	N/A	Dephosphorylation	10	**100%**	0	**0%**	0	**0%**	10
									
Table 7B (Ds) List of rejected terms by the curator in disease axis									
**Unique concept**	**Proposed concept**	**Proposed synonym**	**Rejected**		**Modified**		**Accepted**		**Total**
C2991	D or Disorder	Condition	148	**89%**	11	**7%**	8	**5%**	167
C3262	Neoplasm	Tumor	100	**66%**	12	**8%**	39	**26%**	151
C45576	N/A	Mutation	90	**100%**	0	**0%**	0	**0%**	90
C9305	Malignant neoplasm	Cancer	90	**74%**	2	**2%**	29	**24%**	121
C3114	Hypersensitivity	Sensitivity	50	**94%**	1	**2%**	2	**4%**	53
C3137	Inflammation	Inflammation	49	**73%**	1	**1%**	17	**25%**	67
C18264	Pathogenesis	Pathogenesis	46	**96%**	1	**2%**	1	**2%**	48
C120860	N/A	Accumulation	43	**100%**	0	**0%**	0	**0%**	43
C18078	Carcinogenesis	Tumorigenesis	36	**71%**	4	**8%**	11	**22%**	51
C26845	Parkinson’s D	Parkinson’s D	33	**79%**	0	**0%**	9	**21%**	42
C19296	N/A	Deletion	32	**100%**	0	**0%**	0	**0%**	32
C50753	N/A	Staining	30	**100%**	0	**0%**	0	**0%**	30
C3324	Peutz–Jeghers syndrome	Peutz–Jeghers syndrome	29	**83%**	0	**0%**	6	**17%**	35
C14339	N/A	Knockout mice	27	**100%**	0	**0%**	0	**0%**	27
C20200	N/A	Outcome	26	**100%**	0	**0%**	0	**0%**	26
C45581	Gene amplification abnormality	Amplification	26	**96%**	0	**0%**	1	**4%**	27
C3671	N/A	Injury	25	**86%**	4	**14%**	0	**0%**	29
C53802	Adverse event associated with the gastrointestinal system	Gastrointestinal	25	**83%**	0	**0%**	5	**17%**	30
C42077	Cellular infiltrate	Infiltration	24	**89%**	0	**0%**	3	**11%**	27
C17666	N/A	Germline mutations	23	**100%**	0	**0%**	0	**0%**	23
C75004	Invasion	Invasion	22	**79%**	1	**4%**	5	**18%**	28
C55998	N/A	Platelets	19	**100%**	0	**0%**	0	**0%**	19
C3161	Leukemia	Leukemia	19	**79%**	0	**0%**	5	**21%**	24
C53791	Adverse event associated with infection	Infection	18	**51%**	14	**40%**	3	**9%**	35
C54685	Tissue adhesion	Adhesion	17	**94%**	0	**0%**	1	**6%**	18
C94604	N/A	Mouse model	16	**100%**	0	**0%**	0	**0%**	16
C39723	Immune system finding	Immune system	16	**94%**	0	**0%**	1	**6%**	17
C19987	Cancer progression	Cancer progression	16	**89%**	0	**0%**	2	**11%**	18
C4089	Polyposis	Polyposis	16	**89%**	1	**6%**	1	**6%**	18
C93210	Inflammatory disorder	Inflammatory Ds	16	**76%**	0	**0%**	5	**24%**	21
C19151	Metastasis	Metastases	16	**36%**	5	**11%**	24	**53%**	45
C53809	Adverse event associated with the vascular system	Vascular	15	**88%**	0	**0%**	2	**12%**	17
C17609	Tumor progression	Tumor progression	15	**83%**	0	**0%**	3	**17%**	18
C3208	Lymphoma	Lymphoma	15	**68%**	0	**0%**	7	**32%**	22
C16897	N/A	Necrosis	14	**100%**	0	**0%**	0	**0%**	14
C27990	Toxicity	Toxicity	14	**93%**	0	**0%**	1	**7%**	15
C36117	Invasive lesion	Invasive	14	**70%**	2	**10%**	4	**20%**	20
C62200	N/A	Point mutation	13	**100%**	0	**0%**	0	**0%**	13
C39725	Immunodeficiency	Immunodeficient	13	**93%**	0	**0%**	1	**7%**	14
C120867	N/A	Bacteria	13	**72%**	5	**28%**	0	**0%**	18
C102283	N/A	Extracted	12	**100%**	0	**0%**	0	**0%**	12
C17354	N/A	Frameshift mutation	12	**100%**	0	**0%**	0	**0%**	12
C28193	N/A	Syndrome	12	**100%**	0	**0%**	0	**0%**	12
C2873	N/A	Aneuploidy	12	**100%**	0	**0%**	0	**0%**	12
C45582	N/A	Duplication	12	**100%**	0	**0%**	0	**0%**	12
C18016	Loss of heterozygosity	Allelic loss	12	**92%**	0	**0%**	1	**8%**	13
C14174	N/A	Metastatic	12	**86%**	2	**14%**	0	**0%**	14
C50774	Tissue degeneration	Degeneration	12	**86%**	0	**0%**	2	**14%**	14
C2916	Carcinoma	Carcinomas	12	**80%**	2	**13%**	1	**7%**	15
C3340	Polyp	Polyps	12	**75%**	1	**6%**	3	**19%**	16
C2950	Cytogenetic abnormality	Chromosomal aberration	11	**92%**	0	**0%**	1	**8%**	12
C3117	Hypertension	Hypertension	11	**73%**	0	**0%**	4	**27%**	15
C4872	Breast carcinoma	Breast carcinomas	11	**39%**	0	**0%**	17	**61%**	28
C120945	N/A	Inclusions	10	**100%**	0	**0%**	0	**0%**	10
C17212	N/A	Cell transformation	10	**100%**	0	**0%**	0	**0%**	10
C18133	N/A	Missense mutations	10	**100%**	0	**0%**	0	**0%**	10
C3101	N/A	Inherited D	10	**100%**	0	**0%**	0	**0%**	10
C3174	N/A	Chronic myelogenous leukemia	10	**100%**	0	**0%**	0	**0%**	10
C48189	N/A	Genome instability	10	**100%**	0	**0%**	0	**0%**	10
C48275	N/A	Fatal	10	**100%**	0	**0%**	0	**0%**	10
C8509	Primary neoplasm	Primary tumor	10	**71%**	0	**0%**	4	**29%**	14

Terms always rejected are highlighted in grey. The list is limited to terms proposed at least 30 times by the system. The proposed label does not necessarily correspond to the primary class label; it may be the term synonym identified by neXtA_5_.

A few concepts considered by the annotators (~4%) were chosen from terms not indexed by the name entity recognition module. This minor inconsistency from the input may have contributed to some discrepancy in the results between the manual and neXtA_5_ annotations.

### Highly rejected terms

We have also noticed for both axes that certain terms are frequently rejected, while others are always rejected (highlighted terms; Table 7, Supplementary Data Table 2). Those include synonyms with multiple semantic meanings (formation, growth etc.), terms that are too vague (signaling, signal transduction, regulation, developmental process, carcinogenesis, tumor, autoimmune D, genome instability, outcome etc.), technical terms (RNA interference, RNAi, knockout mice, staining etc.) or non-relevant terms for the axis of interest (such as pathogenesis, memory, methylation, phosphorylation, dephosphorylation, localization, point mutation, accumulation, sensitivity etc.). One possible approach to alleviate this problem would be to put these terms on a black list and not propose them as annotations. Ideally, those terms would also be excluded from the prioritization step, which would also have the advantage of improving the triage step.

### Improvements to the user interface

In addition to improving the document triage and concept extraction algorithms, the users have noticed several improvements to the user interface that would facilitate the workflow.

In the current neXtA_5_ user interface, annotations are displayed according to the position of the descriptor in the text. This was one of the initial specifications of the project, to improve readability and allow curators to know exactly where concepts were extracted from the text. However, while neXtA_5_ is able to suggest relevant descriptors, those descriptors are spread over many irrelevant or trivial descriptors. After performing the usability study, we realized that being able to rank the evidences could deliver a complementary view. In the current GUI, the two types of views are available and the default remains the linear view, which seems somehow more intuitive. We do consider that such complementary revisions are somehow expected as outcome of usability studies.

It would therefore be much more efficient from an interaction point of view to display annotations based on their estimated relevance. We have experimented with improvement to the ranking function of the specific axes. The impact on the performance resulting from these changes in the ranking function seems promising. This additional assessment was performed using TREC_EVAL tool (38), and the results relate the relevance of the annotations proposed by the system at top ranks (P0 for the precision at first rank and P5 for the precision on the five first descriptors returned).

For GO BP, we used a machine-learning approach to improve the ranking of the annotations displayed by neXtA_5_. We used GOCat, a large multiclass multilabel categorizer (39), that exploits more than 100 000 curated citations from the Gene Ontology Annotation (GOA) database (https://www.ebi.ac.uk/GOA/downloads) and aims at inferring GO annotations for any textual input (abstracts, sentences etc.) it receives. As GOCat learns from GOA, the proposed GO concepts are modeling a manual curation task. The GOCat system showed highly competitive results during the BioCreative 2014 competition, which explored a GO automatic annotation task (40). In neXtA_5_, GOCat output is used to promote GO descriptors identified in the input text. Thanks to GOCat, neXtA_5_ improves performances from 0.48 to 0.63 in P0 (+31%) and from 0.28 to 0.35 for P5 (+25%) (Table 8).

**Table 8:** Results of learning to rank applied to annotations

	**Baseline**		**Re-ranking**	
	**P0**	**P5**	**P0**	**P5**
**BPs**	0.48	0.28	0.63	0.35
**Ds**	0.48	0.17	0.59	0.22

For Ds, we used a simple TF–IDF scoring function to estimate the importance of every single annotation. The basic assumption is that important concepts from the curator perspective tend to occur repeatedly in the corpus of texts (i.e. the meaningful entities detected by neXtA_5_ would be repeated through the abstracts). However, these high-frequency concepts may also be regular English words; therefore, the raw frequency of occurrence must be balanced by the inverse document frequency, i.e. the frequency of the concept in a large sample of MEDLINE. As presented in the Table 8, this simple approach results in a significant precision gain of +23% at first rank.

## Perspective

Our results show that the neXtA_5_ system performs well enough to improve the manual curation workflow. The average consensus between curators covers 60% of the concepts for the BPs and 87% for the Ds. neXtA_5_ is then intended to reach similar performance, and prior experiments already show that the exclusion of specific terms and the re-ranking of annotations highly impact on its precision without negatively impacting the recall.

### From abstracts to full-text articles

neXtA_5_ was developed and optimized using abstracts. The analysis of full-text articles is necessary for this tool to be usable in a production setting. Full-text papers pose many problems (3), most are only available in pdf format that should be Optical character recognition (ORC) preprocessed and some are not even available due to the journal access policy. Certain sections, most notably the introduction and the discussion, have a lower interest for the curation, respectively due to the type of information and to the redundancy. For these reasons, the abstract is probably the most useful part of a research article to perform article prioritization. This is not the case for concept extraction; the neXtProt curators (as well as most other curated databases) extract data directly based on experimental results, so it is mandatory that the full text of the paper be reviewed. One middle-way solution would be to allow curators to paste text in a form, which would then be used for concept extraction from neXtA_5_. This would avoid the problem of automatic recognition of article’s sections that is notoriously difficult (41) while making use of the strengths of the system to recognize concepts.

### Perspectives for neXtA_5_

The results of this study convinced us that neXtA_5_ is a valuable addition to our curation pipeline, and we are in the process of implementing neXtA_5_ in the BioEditor curation tool. We are now considering the customization of the curation-support platform to support other use cases of other manually curated resources, such as the detection of positional information (post-translational modifications and variants). These use cases focus more heavily on triage that is both the most mature component of the platform and the most needed service for professional curators. Further developments are ongoing to apply the system to a wider range of curated databases, including core resources of Elixir (https://www.elixir-europe.org/) such as DisProt (42), that will require developing text-mining services to recognize lesser studied entities such as sequence positions. The annotation services will also be expanded to support the annotation of full-text contents. Indeed, while triage is performed mostly on abstracts, the authoring of curated annotations does require the use of full-text contents.

Finally, we are committed to develop neXtA_5_ according to state-of-the-art methodologies. Our work (29) and that of other groups (43) indicate that machine-learning assisted triage method could improve the document retrieval process, outperforming manual curators at least for specific tasks. As machine learning does better than other strategies only in cases where the available body of data is sufficiently large, this approach is currently limited to few data types. We will continue to explore all appropriate algorithms for our use cases and adjust our algorithms as new development occurs that could justify changes in strategies.

## Software availability

A demo version of neXtA_5_ is available at http://candy.hesge.ch/nextA5. The manual judgements on which this study is based are included in Table 3.

## Supplementary Material

Supplementary Data

## Notes

**Database URL:** http://candy.hesge.ch/nextA5; https://nextprot.org
